# Polyethyleneimine impregnated alginate capsule as a high capacity sorbent for the recovery of monovalent and trivalent gold

**DOI:** 10.1038/s41598-021-97228-0

**Published:** 2021-09-08

**Authors:** Yub Raj Dangi, John Kwame Bediako, Xiaoyu Lin, Jong-Won Choi, Che-Ryong Lim, Myung-Hee Song, Minhee Han, Yeoung-Sang Yun

**Affiliations:** 1 Division of Semiconductor and Chemical Engineering, Jeonbuk National University (Formerly Chonbuk National University), Jeonju, Jeonbuk 54896 South Korea; 2 School of Engineering Sciences, University of Ghana, Legon, Ghana; 3 Department of Chemistry, Tri-Chandra Multiple Campus, Tribhuvan University, Kathmandu, Nepal

**Keywords:** Environmental sciences, Chemistry, Engineering

## Abstract

For the first time, a polyethyleneimine-impregnated alginate capsule (PEIIAC) with a high adsorption capacity is developed for the recovery of monovalent and trivalent gold from an acidic solution. The strategy results in a new type of adsorbent, polyethyleneimine impregnated alginate capsule (PEIIAC) with a core–shell structure having a large number of amine groups as cationic binding site, facilitating maximum uptake of anionic auric chloride. The maximum uptake of PEIIAC was 3078 and 929 mg/g for Au (III) and Au (I), respectively, are recordable compared to other reported adsorbents to date. The as-prepared material was executed to check the sorption efficacy for Au (III) and Au (I) in the pH range of 1–12. With an increment in pH, the uptake capacity for Au (III) increased, while the uptake capacity for Au (I) decreased. The FTIR, XRD, and XPS studies revealed that the gold adsorption mechanism includes ionic interactions and reduction, wherein the amine, hydroxyl, and carboxyl groups are involved. The capsule showed a higher adsorption efficiency than other reported sorbents, making the material applicable in acidic solutions for the recovery of Au (I) and Au (III).

## Introduction

Precious metals (PMs) are mainly used in electronics owing to their high electrical conductivity and anticorrosive nature^1,2^. Owing to the rapid replacement of the electronic devices, a large quantity of electronic waste (e-waste) is discharged into the environment^3,4^. Currently, the annual e-waste generation is 40 million metric tons which increases every year^5,6^. A significant amount of gold is present in e-wastes such as Computer, Cell Phones, Tablets, Television, Camera, Media Players, Stereos/Radios, Game Consoles/Accessories and so on^7,8^. For instance, 200 g of gold can be extracted from one ton of PCB waste. In 2016, approximately 20% of e-waste was recycled out of the 45 million tons. Approximately 10 billion euros of gold is lost in the form of e-waste^9,10^. Nearly 200 g of gold per ton of scrap can be extracted from e-waste, while gold ores contain 5–30 g of gold per ton^11^. Therefore, urban mining from e-waste costs lower than virgin mining (limited natural reserves). Additionally, a very low concentration of gold is found in oceans (> 20 million tons), freshwater (< 10 µg/L), and wastewater (< 10 µg/L). Annually, about 1.5 million euros of gold is lost as sewage in Switzerland and the UK^2^. Therefore, gold recovery from e-waste and wastewater is crucial for saving the economy and the environment. Gold can be of monovalent and/or trivalent forms in the solution phases.

Presently, several technologies such as adsorption, solvent extraction, membrane separation, chemical precipitation, and ion exchange have been applied for the recovery of gold recovery from the leaching solutions^12–16^. Among them, the adsorption method has been widely applied because of its sustainability, promising, simplicity, eco-friendliness, low operating cost, and high efficiency^17,18^. Additionally, adsorbents can be reused several times because of the reversibility of the adsorption process. Several adsorbents used for gold adsorption include biosorbent, synthetic polymers, porous carbon, and metal-based adsorbents. However, existing sorbents have fewer surface functional groups, low sorption capacities, and are unstable in acidic solutions^3^. To overcome these problems, developing multifunctional, high sorption capacity, and acid-stable adsorbents for the recovery of gold is crucial.

Biopolymers such as alginate, chitosan, starch, cellulose, pectin, carboxymethyl cellulose, and protein, have gained more attention as precursors for the development of adsorbents with multifunctionality, natural abundance, biocompatibility, biodegradability, and nontoxicity^19^. However, raw biopolymers have a low metal adsorption capacity compared to their derivatives^20^. Several physical and chemical modifications have been studied to enhance the binding sites through the addition of functional groups and porosity. The adsorption capacity of raw biopolymers can be enhanced by increasing the number of binding sites through functionalization and gel formation. Excess amine and thiol functional group-containing molecules have been applied for surface coating, cross-linking, and composite formation to enhance adsorbent performance, owing to their high chelation power toward metals^21,22^. Physical modification of biopolymers includes transformation of sol–gel composite into different stable shapes like fiber, bead, capsule, and membrane, which increases porosity and functional group exposure resulting in high sorption capacity. According to Pearson acid–base theory^23^, N of amines and S of thiols acting as hard base due to presence of lone pair of electron has very high affinity toward precious metals as soft acid^24,25^. Based on the hard and soft acids and bases (HSAB) principle, the adsorption capacity of the biopolymer can be increased by the incorporation of a large number of chelators like polyethyleneimine (PEI), amino acids, and polyacrylic acid. Several efforts have been done to introduce chelating groups into biopolymer, examples include Aliquat-366-impregnated alginate capsules^1^, cross-linked cellulose gel by sulfuric acid^26^, and N-aminoguanidine functionalized cellulose powder^27^. Above mentioned adsorbents might solve the selectivity problem, but they lack the significant adsorption capacity required for the recovery of gold. For enhancement of sorption capacity toward Au (III), some reported attempts are PEI-modified bacterial biosorbent fibers^28^ and glutaraldehyde (GA)-PEI-alginate fibers^3^.

Alginate (AG) has huge application in adsorption technology being hydrophilic polysaccharide retaining all properties of biopolymers^1,3,18^. It is found in cell walls of brown algae which forms viscous gum on hydration. It is the most important anionic biopolymer having hydroxyl, carboxyl, and ether functional groups in β-D-mannuronate and α-L-guluronate monomers which are beneficial for the composite formation and chemical transformation. Due to these characteristics, alginate derivatives have been broadly applied for metal ion recovery and removal^29^. However, it does not have a cationic amino group as an anion exchanger. One way to amino functionalization in alginate is to make a composite of alginate blende with amino molecules. To incorporate anion exchanger, alginate has been mostly fabricated with suitable nitrogen-rich molecules through different techniques like cross—linking, surface coating, and impregnation (encapsulation)^30,31^. For example, Aliquat-336 impregnated alginate capsule was an effective sorbent for selective recovery of gold (192 mg/g)^32^. Recently, GA-PEI-alginate fiber was developed for high adsorption of gold (2300 mg/g)^3^. Many attempts have been done to prepare potential alginate-based sorbents. However, very little focus has been given to develop acid-stable alginate-based adsorbents with very high adsorption capacity.

This study provides a way toward a novel sorbent able to recover precious metals like gold with different valences. In this study, for the first time, we prepared PEIIAC using an impregnation method as a high-capacity sorbent for mono and trivalent gold recovery from aqueous system. Although PEI is a good chelating and reducing agent for gold, it is water-soluble and cannot be used as an adsorbent. Therefore, PEI should be immobilized in a certain way. In its study, therefore, PEI was impregnated within the alginate shell. CMC was used as an anionic polymer to disperse and stabilize PEI as a cationic polymer inside the calcium alginate shell as an interlocking matrix support. PEI was selected as a chelating agent for penetrating gold ions because of the binding sites that are provided with three types of nitrogen including primary, secondary, and tertiary amines in the molecule, which could be utilized as binding sites. Primary, secondary, and tertiary amines of PEI as cationic sites can interact with anionic carboxylates and the hydroxyl groups of biopolymers such as alginate through electrostatic attraction. This facile strategy formed an acid-stable novel cationic PEIIAC composite capsule with a high adsorption capacity for anionic auric chloride and aurocyanide. The composite PEIIAC capsule proved as high capacity sorbent by adsorbing an outstanding auric chloride uptake of 3078 mg/g, which is the highest recordable value reported to date. The effects of pH, sorption kinetics, and sorption isotherms were studied to determine the adsorption performance of the capsule. The morphology of the capsule was studied by microscopy. The mechanism of gold adsorption and reduction of the capsule was studied using Fourier transform infrared (FT-IR) spectroscopy, X-ray diffraction (XRD), and X-ray photoelectron spectroscopy (XPS) before and after sorption.

## Materials and methods

### Materials

Sodium alginate (Showa Chemical Industry Co., Ltd., Japan) as an anionic biopolymer was used to make the capsules. Molecular weight of Sodium carboxymethyl cellulose (CMC) with high viscosity was approximately 700 kDa, which was used in the capsule. CMC (Sigma-Aldrich, Korea) was used as an anionic biopolymer to disperse PEI. Branched PEI as a cationic polymer (average M_W_: 750,000 by light scattering and concentration: 50 wt % in H_2_O) was purchased from Habjung Moolsan Co. Ltd. (Korea). Calcium chloride (CaCl_2_.2H_2_O, Samchun Pure Chemical Co., Ltd., Korea) was used as a divalent cross-linker to prepare the alginate capsules. Gold solutions were prepared by dissolving hydrogen tetrachloroaurate (III) hydrate (HAuCl_4_.3.6 H_2_O, Kojima Chemicals Co., Ltd., Japan) in 0.1 M HCl and potassium dicyanoaurate (I) K [Au (CN)_2_], Kojima Chemicals Co., Ltd., Japan) in double-distilled water (DW). All analytical-grade chemicals were used for the experiments.

### Preparation of the PEIIAC composite capsules, Au-alginate, Au–PEI and Au-CMC

**Figure 1:**
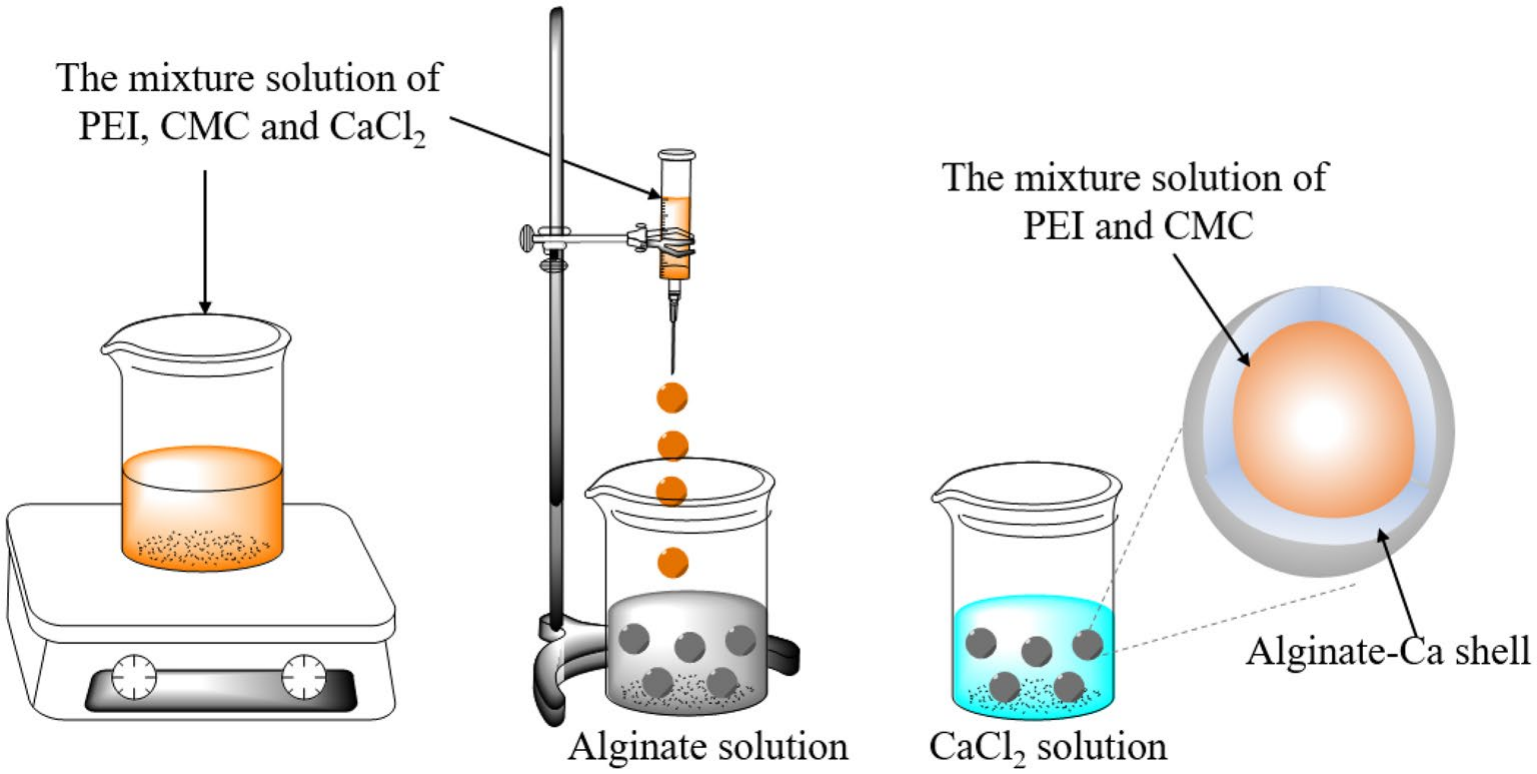
Schematic diagram for the preparation of PEI-Alginate capsule.

The preparation of the PEIIAC composite capsule is shown in Fig. 1. 3 g of CMC and 5 g of polyethyleneimine were separately dissolved in 50 mL of 2% calcium chloride solution with magnetic stirring for 24 h. Subsequently, they were mixed just before the formation of the capsule and stirred with a magnetic stirrer to obtain a homogeneous mixture. The viscous mixture of CMC and PEI in CaCl_2_ was added to 200 mL of 0.4% sodium alginate solution through a syringe. Capsules were created when the Ca^2+^ ions of the drops came into contact with the alginate. The capsules were then cured in 200 mL of 2% CaCl_2_ solution for 24 h, washed with distilled water several times, and stored in DW at room temperature for further use. The capsules were stored in deionized water for 60 days during experiment. The calcium alginate biopolymer did not develop mold with storage in deionized water at room temperature. Wet capsules were used as sorbents during the experiment.

Sample was prepared to study and compare the interaction of Au (III) and Au (I) with alginate, CMC, and PEI. 50 mL of PEI, CMC, and alginate solutions were prepared separately by dissolving 1 g of each molecule in DW under continuous magnetic stirring for 3 h. After then, 50 ml of 1000 ppm of Au (III) and Au (I) solutions were separately mixed with before mentioned solutions. Later, 100 ml of each solution were kept in a shaking incubator with 120 rpm and 25 °C for 24 h. After then the viscous samples solutions were frozen at − 40 °C for 3 h to get solid, which was freeze-dried for 1 week. The resultant sample was used for FTIR analysis.

### Characterization of the capsules, PEI, CMC and alginate before and after adsorption of gold

The prepared capsules were characterized before and after the adsorption of gold. Light microscopy was used to study the physical appearance of capsules kept in a Petri dish containing water. Dry capsules were powdered and used for FTIR, DRX and XPS analysis. The functional groups of the capsules (before and after adsorption) were identified using FT-IR spectroscopy in the range of 4000–400 cm^−1^, a PerkinElmer spectrophotometer (Spectrum GX, FTIR System), and a KBr disk. The mixture containing 1/8″ of the solid sample and 0.25–0.50 teaspoons pf KBr was grinded with pestle to prepare KBr disk. The crystallinity of the pristine and gold-loaded capsules was determined using an X-ray diffractometer (XRD, X’pert powder, PANalytical, The Netherlands). The surface chemistry of the capsule before and after the sorption of gold was studied using X-ray photoelectron spectroscopy (XPS).The analysis of atomic valence states of N, Au, Cl and O was carried out with the help of an AXIS-NOVA spectrometer (Kratos Analytical, Ltd., UK) with monochromatic Al Kα as the X-ray source (1486.71 eV of photons).

### Dry weight experiment

Experimental calculations were conducted based on the dry weight of the sorbent to understand the actual uptake of metals. Wet capsules were used in the experiment, considering the probability of blockage of the active sites in the dry capsules. A dry weight experiment was conducted to determine the water content of PEIIAC. 0.4 g of wet weight of capsules were kept in a freeze drier to sublimate the water of the sorbent for 72 h. The freeze dryer equipment (FD-Series, South Korea) are used at − 40 °C and 96.30 mTorr. Dry weight percentage was calculated using the following equation:

1 $$\mathrm{Dry \,weight \,ratio }(\mathrm{\%}) =\frac{\mathrm{Adsorbent\, dry \,weight}}{\mathrm{Adsorbent\, wet\, weight}} \times 100$$

### Adsorption experiments

In adsorption studies, the effect of pH, isotherms, and kinetics experiments were conducted by keeping 0.4 g of wet capsules in 30 ml of a gold solution in 50 ml falcon tubes and placed in a multi-shaking incubator at 25 °C for 24 h under 120 rpm speed. In pH effect, NaOH and HCl of different molar concentrations were used to maintain required pH 1–12 values. Initial concentration of 1000 ppm of both Au (III) and Au (I) were used to carry out pH edge. An inductively coupled plasma-atomic emission spectrometer (ICP-AES, ICPS-7510 Shimadzu, Japan) was used to calculate the remaining gold in the supernatant solution after dilutions. Gold solutions of 50–5500 mg/L were used for the isotherm experiments. The kinetics experiments were conducted at various time ranges. The adsorption capacity of the capsules was calculated using the following equation:

2 $$q=\frac{\left({C}_{0}-{C}_{e}\right)V}{M}$$

where, ${C}_{0}$ and ${C}_{e}$ are the initial and equilibrium concentrations (mg/L), respectively, V is the volume (L), and M is the dry mass of the adsorbent (g).

## Results and discussion

### Effect of pH

**Figure 2:**
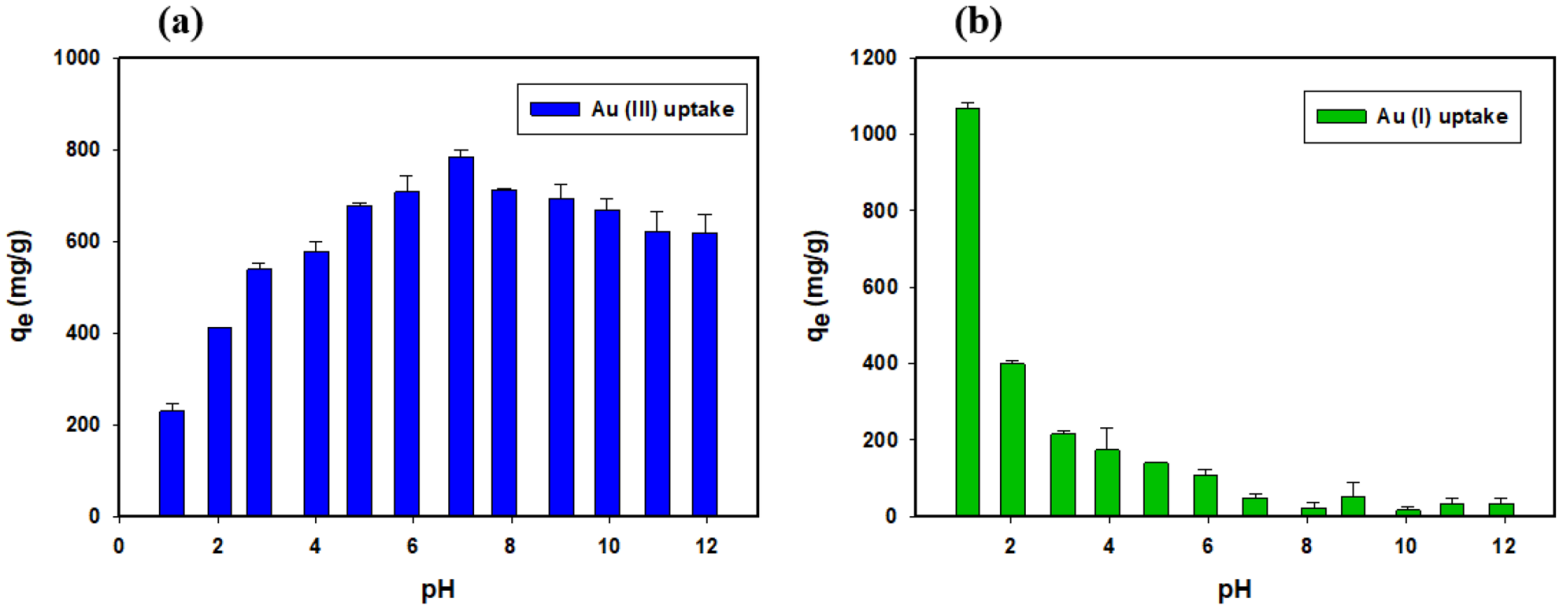
Effect of pH for adsorption of (**a**) Au (III) and (**b**) Au (I) into the capsule (C_0_ = 1000 mg/L, pH = 1–12, t = 24 h, M_ads_/V_sol_ = 0.4 g/30 mL, T = 25 ± 2 °C).

The surface functional group of adsorbents and chemical speciation of adsorbate affected by the pH of the solution. So, it is crucial to study the effect of pH at all range on the adsorption of ionic species competing with H^+^ and OH^−^ ions in an aqueous solution. The adsorption trend of Au (III) and Au (I) onto PEIIAC at all pH ranges are shown in Fig. 2a,b, respectively. In the case of Au (III), sorption of it increases from 1 to 14 of pH due to adsorption and precipitation. According to gold speciation, [AuCl_4_]^−^ exists at pH 1–4 and Au (OH)_3_ at pH 4–14^33^. Precipitation of Au (III) starts from pH4. The high adsorption at pH 4–14 is not due to adsorption but it is due to precipitation of gold in the form of Au (OH)_3_ . The capsules become positively charged due to protonation in an acidic solution resulting in electrostatic attractions between [AuCl_4_]^−^ and the capsules and anion exchange between [AuCl_4_]^−^ and Cl^−^. So, the optimum pH for Au (III) recovery was 1–4 which is supported by literature data^34–39^. Recovery of Au (I) increased with the decrease in pH for the capsule. At pH 1–7, Au (I) precipitation is possible in the form of Au (0). So, it looks very high adsorption value at pH1 which is actually due to the precipitation of gold. At pH 7–14, adsorption is low since capsules become negatively charged resulting in repulsion with anionic species. The high stability and elastic nature of the capsule is due to crosslinking with ${Ca}^{2+}$ and intermolecular hydrogen bonding between –COOH groups at low pH^40^. Adsorption mechanism concerning PEIIAC and [AuCl_4_] ^−^ can be expressed as Eq. (3).

### Sorption kinetics

Sorption kinetics is important in industrial applications for process design and operation. The pseudo-first-order^41^ and pseudo-second-order kinetic equations were used to study the kinetics of Au (III) and Au (I) adsorption on PEIAC which are expressed as below:

3 $$\mathrm{Pseudo}-\mathrm{first}-\mathrm{order model}: {q}_{t}={q}_{1}(1-\mathrm{ exp}(-{k}_{1}\mathrm{t}))$$

4 $${\mathrm{Pseudo}-\mathrm{second}-\mathrm{order model}: q}_{t}=\frac{{q}_{2}^{2} {k}_{2}\mathrm{t}}{1+{{q}_{2}k}_{2}\mathrm{t}}$$

where: q_1_ and q_2_ are amounts of metal ions adsorbed at equilibrium, q_t_ is amount of metal ions adsorbed at time t, k_1_ and k_2_ are rates constant for Pseudo-first-order adsorption and pseudo-second-order adsorption respectively.

**Figure 3:**
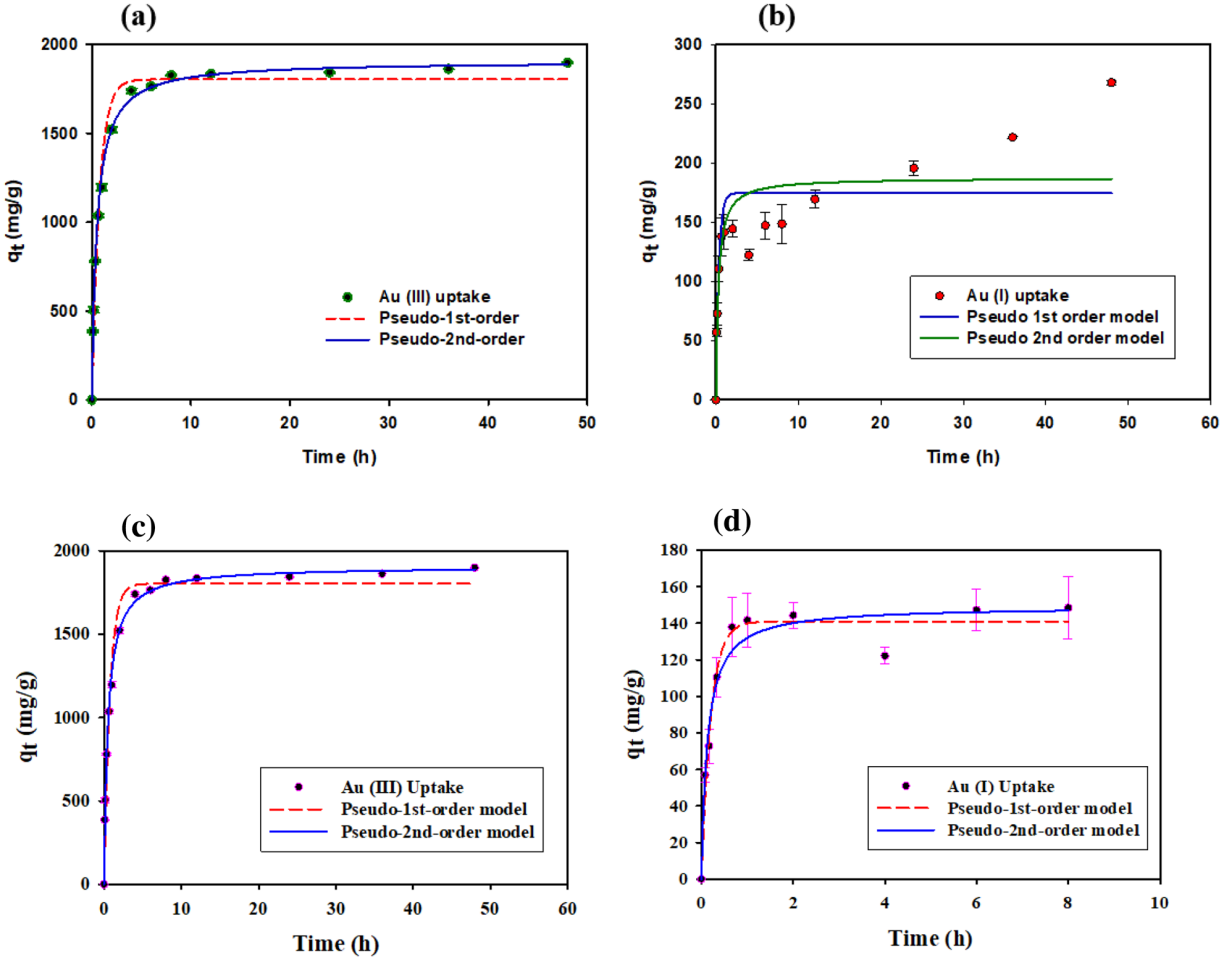
Kinetics of (**a**) Au (III) and (**b**) Au (I) adsorption into the capsule (C_0_ = 1000 mg/L, pH = 2, t = 48 h, M_ads_/V_sol_ = 0.4 g/30 mL, T = 25 ± 2 °C).

According to regression coefficient (R^2^) and constant parameters as shown in Table 1, experimental data were best fitted for pseudo-second-order kinetic model in case of both oxidation state of gold^42^. According to kinetics graph shown in Fig. 3a,b, adsorption increases initially with time due to the presence of more sorption sites on capsules and reached equilibrium state within 8 h in the case of Au (III) and 4 h in the case of Au (I). There is repulsion between free carboxylate anion of alginate and anionic auric chloride and aurocyanide. Carboxylate anion of alginate are crosslinked with calcium ion to form calcium alginate shell. Compacted calcium alginate shell of the capsule formed after crosslinking might decrease rate of gold diffusion. Continuous reduction and lowering diffusion of gold with the shell might be the reason for very low kinetics.

**Table 1:** Comparison of the kinetic parameters for the adsorption of Au (I) and Au (III) onto PEIIAC.

Metal ions	Pseudo-first-order			Pseudo-second-order		
	q_1_ (mg/g)	K_1_(min^−1^ )	R^2^	q_2_ (mg/g)	K_2_ × 10^−4^(g/mg min)	R^2^
Au (I)	280.65 (20.06)	3.1996 (1.2343)	0.7117	299.65 (20.64)	0.0134 (0.0063)	0.7730
Au (III)	1803.31 (39.99)	1.3262 (0.1363)	0.9746	1905.71 (20.69)	0.0010 (7.0245)	0.9952

Based on the regression coefficient (R^2^) value, data are well fitted with the pseudo-first-order (R^2^ = 0.9727) and pseudo second-order kinetic model (R^2^ = 0.9587) model for 8 h as shown in the Fig. 3b (2nd figure). Reduction of aurocyanide might be the reason for the increase in adsorption kinetics after 8 h. Gold is known to be easily reduced with PEI^3^ and gold reduction was also confirmed in the study (Fig. 8c). However, the pseudo-first order and pseudo-second order kinetic models do not cover the reduction. That is likely why a larger deviation occurs after 8 h. For a comparative study of Au (III) and Au (I) kinetics at pH2, experimental data of capsules’ sorption kinetics of Au (I) were fitted with the pseudo-first-order and pseudo-second-order models even though the fit was low (R2 < 0.78).

### Sorption isotherm

Au (III) and Au (I) adsorption mechanism on PEIIACs were described by using Freundlich^43^ and Langmuir models which are applicable for heterogeneous and homogeneous surface adsorption respectively. These models can be expressed as below:

5 $$\mathrm{Langmuir\, model}: {q}_{e}=\frac{ {q}_{m}\mathrm{b}{C}_{f}}{1+\mathrm{b}{C}_{f}}$$

6 $${\mathrm{Freundlich\, model}: q}_{e}={k}_{F}{C}_{f}^\frac{1}{n}$$

where, ${q}_{e}$ is the equilibrium amount of adsorbed metal (mg/g), ${q}_{m}$ is the maximum uptake (mg/g), b is the Langmuir equilibrium constant (L/mg), ${C}_{f}$ is the final concentration (mg/L), ${k}_{F}$ is the Freundlich constant (mg/g) (L/g)^1/n^, and n is the Freundlich exponent.

**Figure 4:**
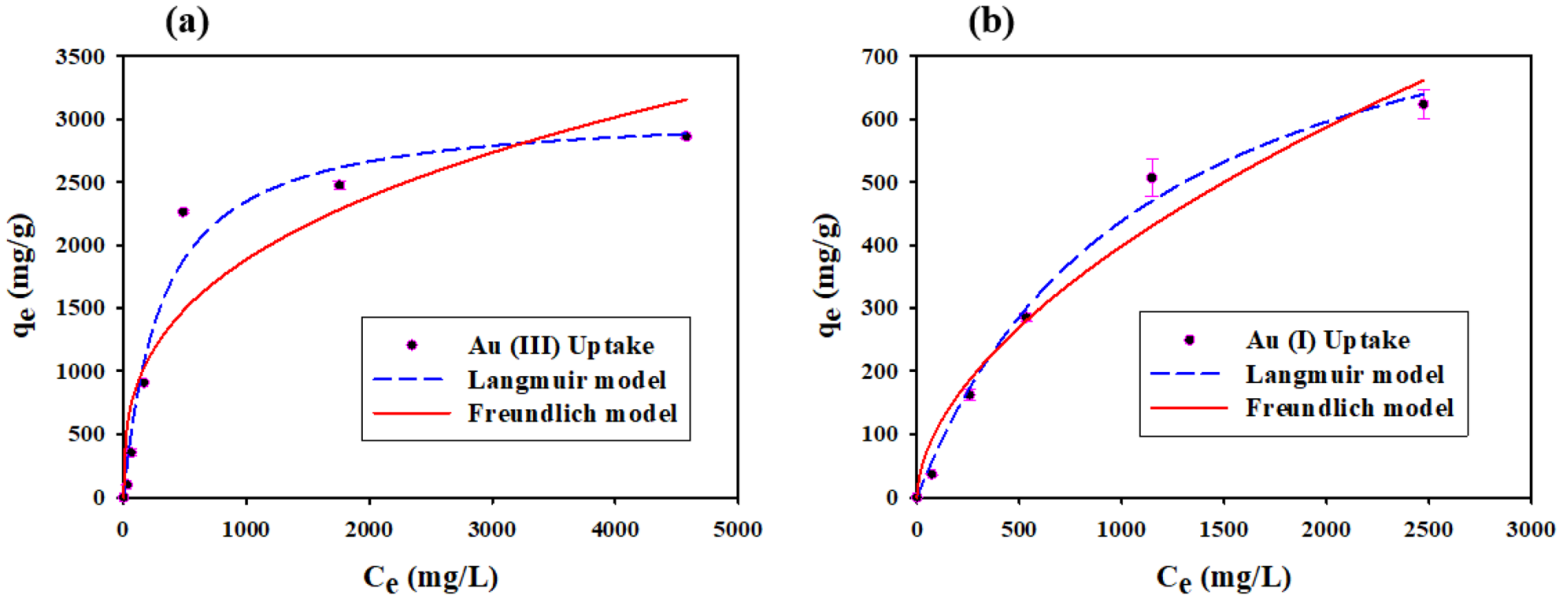
Isotherms of (**a**) Au (III) and (**b**) Au (I) adsorption into the capsule (C_0_ = 0–5500 mg/L, pH = 2, t = 24 h, M_ads_/V_sol_ = 0.4 g/30 mL, T = 25 ± 2 °C).

Isotherms inform maximum uptake of gold from solution at a constant temperature. Adsorption of both gold increased with increasing initial concentration till the equilibrium state was achieved as shown in Fig. 4a,b, respectively. According to the highest regression coefficient (R^2^) value shown in Table 2, the experimental data were best fitted for Langmuir isotherms than Freundlich isotherms in the case of Au (III) and Au (I). The maximum uptake of Au (III) and Au (I) were predicted by the Langmuir model as 3077.56 ± 226.14 mg/g and 928.80 ± 77.71 mg/g respectively. The capsules have well adsorption capacity compared with other materials as shown in Table 3.

**Table 2:** Comparison of the Langmuir and Freundlich parameters for the adsorption of Au (I) and Au (III) onto PEIIAC.

Metal ions	Langmuir model			Freundlich model		
	${\mathrm{q}}_{\mathrm{m}}$(mg/g)	b (L/mg)	R^2^	k_F_ (mg/g)(L/g)^1/n^	n	R^2^
Au (I)	928.80 (77.71)	0.0009 (0.0002)	0.9927	8.3321 (5.1196)	1.7863 (0.2667)	0.9658
Au (III)	3077.52 (226.15)	0.0032 (0.0009)	0.9717	182.5127 (120.7155)	2.9581 (0.7587)	0.8728

Standard errors are present in parentheses.

**Table 3:** Comparison of the maximum uptake capacity for different adsorbents.

Adsorbent and species	q_m_ (mg/g)	References
L-cysteine impregnated alginate capsules	1.51	^18^
Cross-linked chestnut pellicle	2100	^44^
Ca-alginate beads	1.47	^45^
Wattle tannin gel	8000	^46^
Thiourea modified alginate powder	6.40	^47^
Chemically modified chitosan	669.8	^48^
Porous epichlorohydrin / thiourea modified alginate(PETA)	1.97	^49^
Dimethylamine modified persimmon waste gel	1109.11	^50^
Sulphuric acid cross-linked alginate powder	5.64	^26^
Crosslinked persimmon tannin gel	1516.9	^51^
Raw biomass/DCB toward Au(I)	50.19/86.16	^13^
Bisthiourea modified persimmon tannin gel	1020.46	^52^
Rice husk carbon	149.72	^53^
PEIIACs toward Au(III)	3077.52	This work
PEIIACs toward Au(I)	928.80	This work

### Characterization and possible adsorption mechanism

**Figure 5:**
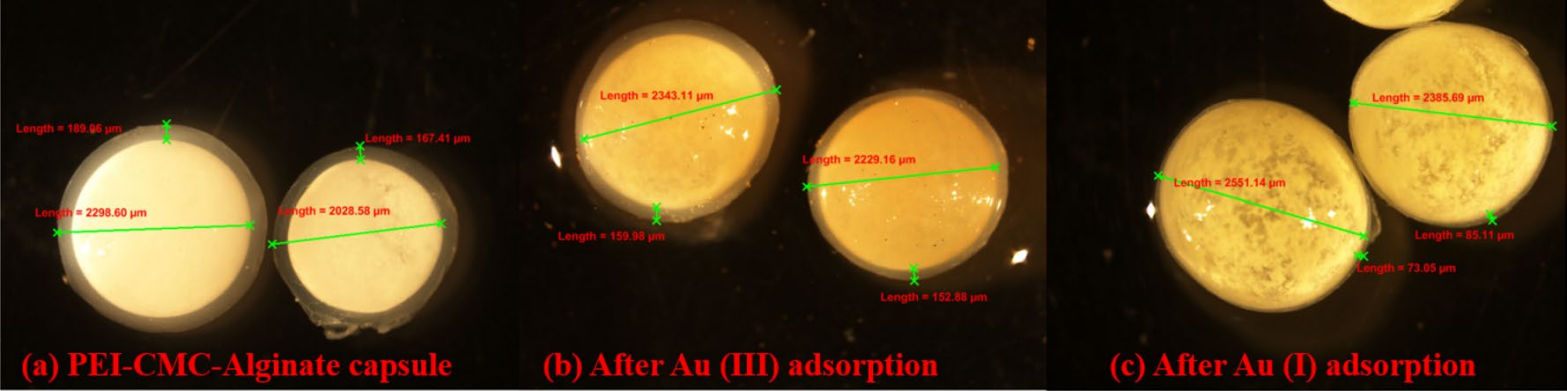
Light Microscopic image of (**a**) PEIIAC, (**b**) after Au (III) adsorption, and (**c**) after Au (I) adsorption.

Visual observation of the capsules before and after adsorption of Au^3+^ and Au^+^, through the microscope is shown in Fig. 5a–c. The average diameter of the nearly spherical capsule was 2.2 ± 0.1 mm. Diameter of several capsules were measured and expressed as average value with standard error. The uncertainty of the diameter measurement of the capsule was 2.2 ± 0.1 mm. After adsorption of gold, the capsules were slightly expanded as shown in Fig. 5b,c. Expansion of the capsule after Au (I) adsorption is higher compared to Au (III) sorption.

**Figure 6:**
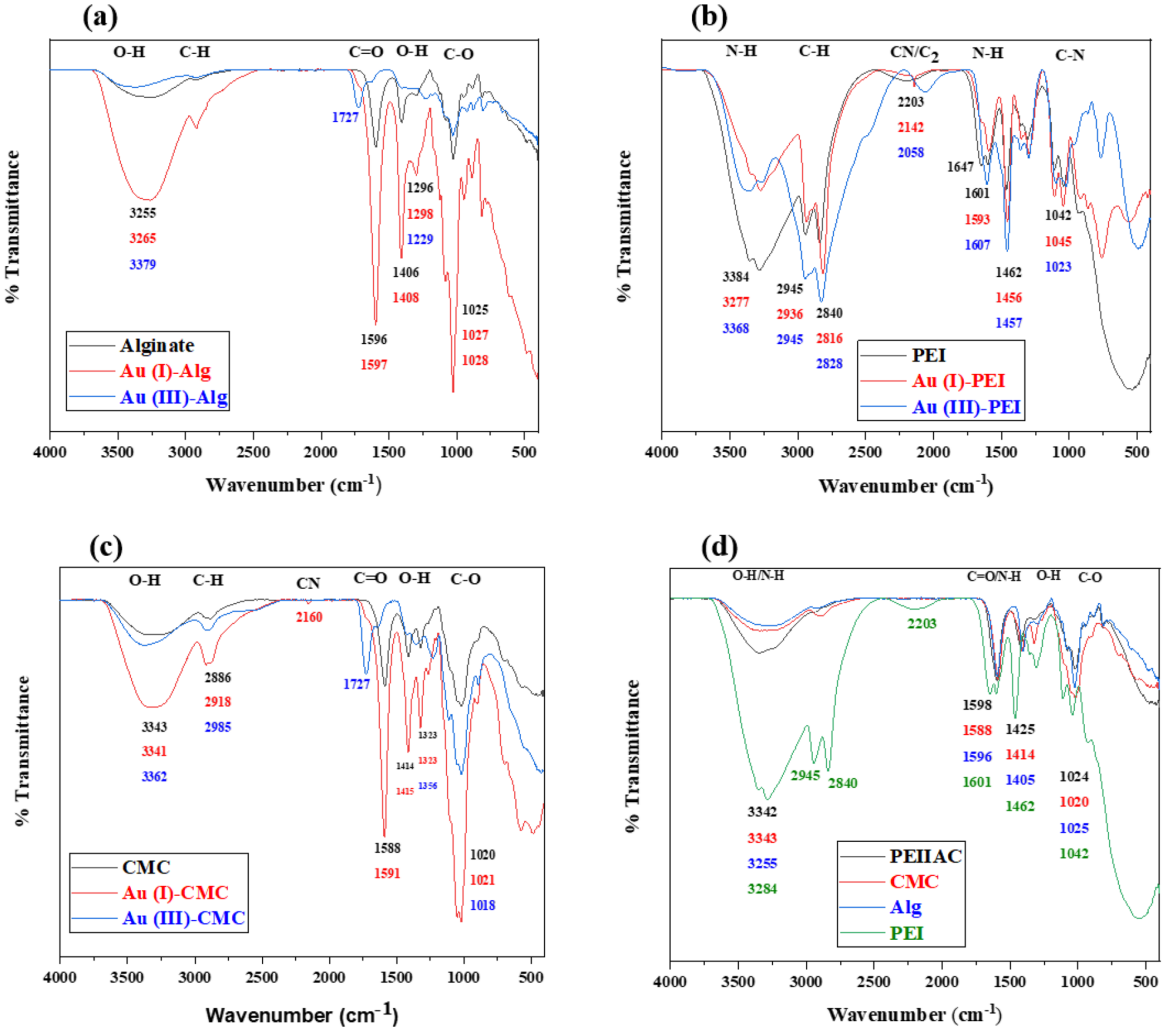
FTIR spectra of (**a**) Au–Alginate, (**b**) Au-PEI, (**c**) Au-CMC, and (**d**) PEIIAC, CMC, Alg and PEI.

Furthermore, the capsule has a white mixture of PEI and CMC surrounded by calcium alginate shells as shown in Fig. 5a. When the viscous mixture of PEI, CMC, and CaCl_2_ is dropped into the alginate solution, Ca +  + ions of the mixture diffuse out, meet alginate, and form alginate gel on the surface of the drop via ionic crosslinking. This leads to a core–shell structure with PEI and CMC in the core and alginate in the shell. To confirm calcium alginate shell formation, FTIR spectra of PEIIAC, CMC, Alginate and PEI were compared as shown in Fig. 6d.The IR band position of the capsule is significantly different compared to those of sodium alginate, PEI, and CMC. The peak of PEIIAC at 3200–3600 cm^−1^ is narrower than those of sodium alginate, PEI, and CMC^3^. The difference is likely due to the involvement of hydroxyl and carboxylate groups in crosslinking with calcium ions during alginate shell formation. As a result, narrow bands of calcium alginate appear due to decreasing the number of O–H groups involved in hydrogen bonding. The shifting of the peak due to carboxylate ion is because of displacement of the sodium ion with calcium ion in sodium alginate.

To study the interaction of Au (III) and Au (I) with alginate, CMC, and PEI, FTIR analysis of the polymers before and after adsorption of gold was carried out as shown in Fig. 6a–c. The difference in spectra and absorbance band before and after adsorption indicate the interaction of respective molecules with gold. Different spectra of Au (I)-polymer and Au (III)-polymer indicate different interaction mechanisms. The appearance of nitrile peak in Au (I)-PEI (2142 cm^−1^) and Au (I)-CMC (2160 cm^−1^) confirms the existence of aurocyanide without reduction. In Fig. 6a,c, tapering of O–H peak and appearance of carbonyl peak at 1727 cm^−1^ after Au (III) adsorption might be due to the reduction of Au (III) with a hydroxyl group^3^. As shown in Fig. 6b, the narrowing of the N–H peak at 3200–3600 cm^−1^ and decrease in absorbance at 1000–1700 cm^−1^ after adsorption of gold indicate electrostatic, chelation, and redox interaction. Different spectra of Au (III)-PEI and Au (I)-PEI indicate different actions of PEI with them. PEI does electrostatic, chelation, and redox interaction toward Au (III) while only electrostatic and chelation action on Au (I).

**Figure 7:**
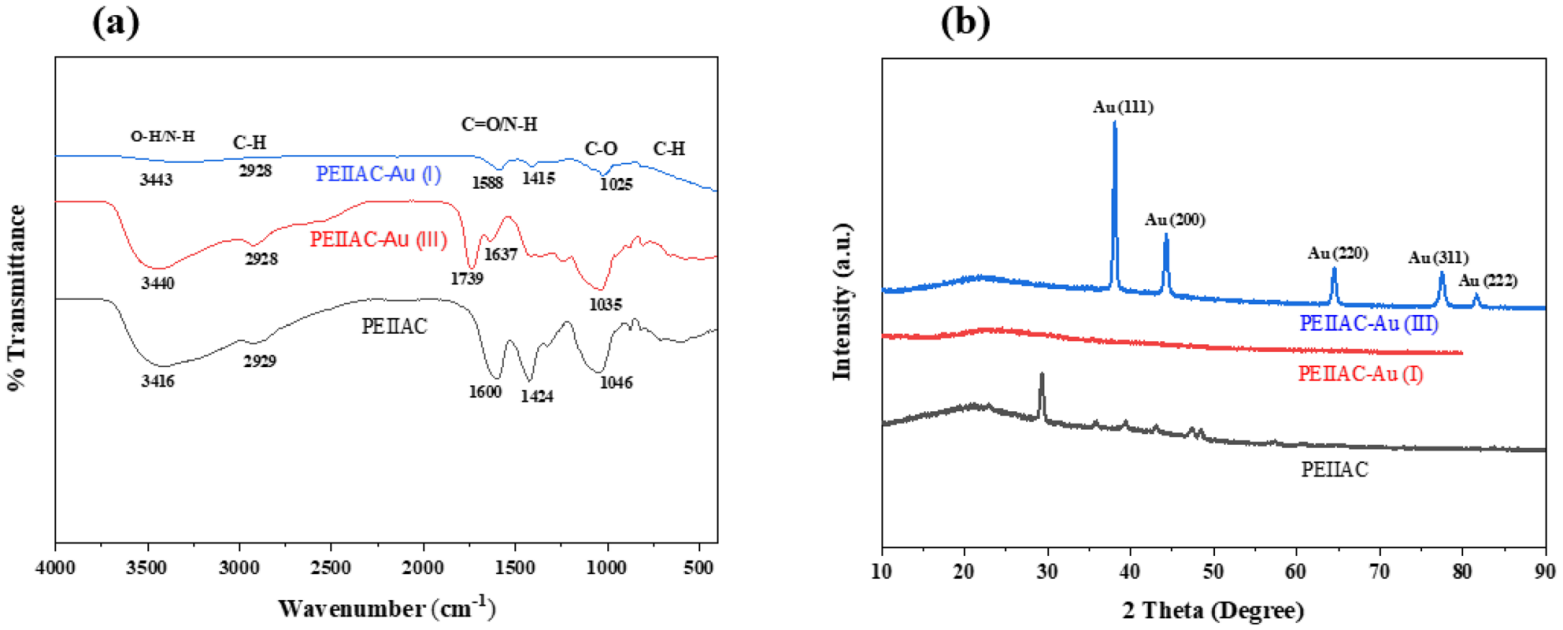
(**a**) FTIR and (**b**) PXRD patterns of PEIIAC before and after Au (III) and Au (I) sorption.

To understand the adsorption mechanism, impregnation of PEI inside capsule and surface functional group, the capsules before and after adsorption of Au^3+^ were characterized by FTIR spectroscopy which is shown in Fig. 7a. It is important for the identification of functional groups that are mainly responsible for adsorption. The functional group and fingerprint region of spectra show characteristics peak of the functional group present in individual PEI, CMC, and Alginate with a slight shift due to interaction during fabrication. The change in peak value after the adsorption of gold indicates interaction between adsorbate and adsorbent through the active site. The O–H and N–H bond stretching vibration of the capsules at 3416 cm^−1^ changes to 3440 cm^−1^ after adsorption of ionic gold^3^. The change in peak value pointed out the interaction between adsorbate and adsorbent through ionic chelating interaction. The broad peak indicates the presence of a large number of hydroxyl and primary amino groups in the composite which reduces after adsorption due to their participation of them in ion exchange and reduction^54^. The C = O of carboxylate salt in alginate and CMC and N–H of PEI show absorption peak at 1600 cm^−1^ which increase to 1637 and 1739 cm^−1^ after the sorption of gold^1^. Such an increase in absorption peak value implies (I) ionic and (II) chelating interaction between adsorbate and adsorbent^55^. The peak at 1424 due to carboxylic acid O–H and alcoholic O–H bending of the capsule shifts to 1417 after the sorption of the gold. It emphasizes ionic interaction between the O–H/N–H functional group of the capsule and the anionic form of gold. The stretching vibration of primary alcohol C-O, C–O–C of polysaccharide and aliphatic C-N present in the capsule occurs at 1046 cm^−1^ and decreases to 1035 cm^−1^ after sorption of adsorbate depicting participation of them in adsorption and reduction^56^.The C-H bending at 877 cm^−1^ of the capsule is mainly due to the aliphatic –CH_2_– group of Alginate, CMC, and PEI, which changes to 881 cm^−1^ after sorption indicating the change in the electronic environment. Furthermore, the peak at 2928 cm^−1^ belongs to aliphatic C-H stretching and bending vibration which reduces to 2927 cm^−1^ after adsorption pointing adsorption of adsorbate onto adsorbent^57^. The shift in FTIR peak after adsorption of gold indicates adsorption of gold with capsule through –NH_2_, –OH, –O– and COOH functional groups, which was not sufficient for confirmation of the reduction of gold inside the capsule. Therefore, XRD was carried out to know metallic gold formation after sorption.

The multipurpose X-ray diffraction (XRD) was conducted to know the crystallinity of the capsules and the mechanism of gold adsorption. As shown in Fig. 7b, the XRD patterns of the capsule before and after adsorption of trivalent gold are different, indicating that the formation of metallic gold after adsorption and reduction. The formation of metallic gold indicates that the possible mechanism was adsorption followed by reduction. The XRD pattern of capsules after adsorption of trivalent gold displayed sharp peaks of Au^0^ at 38.1, 44.3, 64.5, 77.5, 81.6, corresponding to the Bragg’s reflections of (111), (200), (220), (311) and (222), respectively^58^. The results indicate the formation of crystalline metallic gold nanoparticle having face-centered cubic lattice inside the capsule, justifying that ionic Au (III) was adsorbed in PEIIAC followed by reduction. Also, the intense peak at (111) plane supports the formation of reduced gold. Thus, XRD justified the hypothesis that the redox-active PEIAC can reduce trivalent gold of auric chloride to metallic gold at acidic solutions (pH1). However, there is no significant difference in the XRD pattern of the capsule before and after adsorption of monovalent gold in aurocyanide as shown in Fig. 7b. This indicates that there is no reduction of monovalent gold from aurocyanide after adsorption with PEIIAC at the acidic solution.

**Figure 8:**
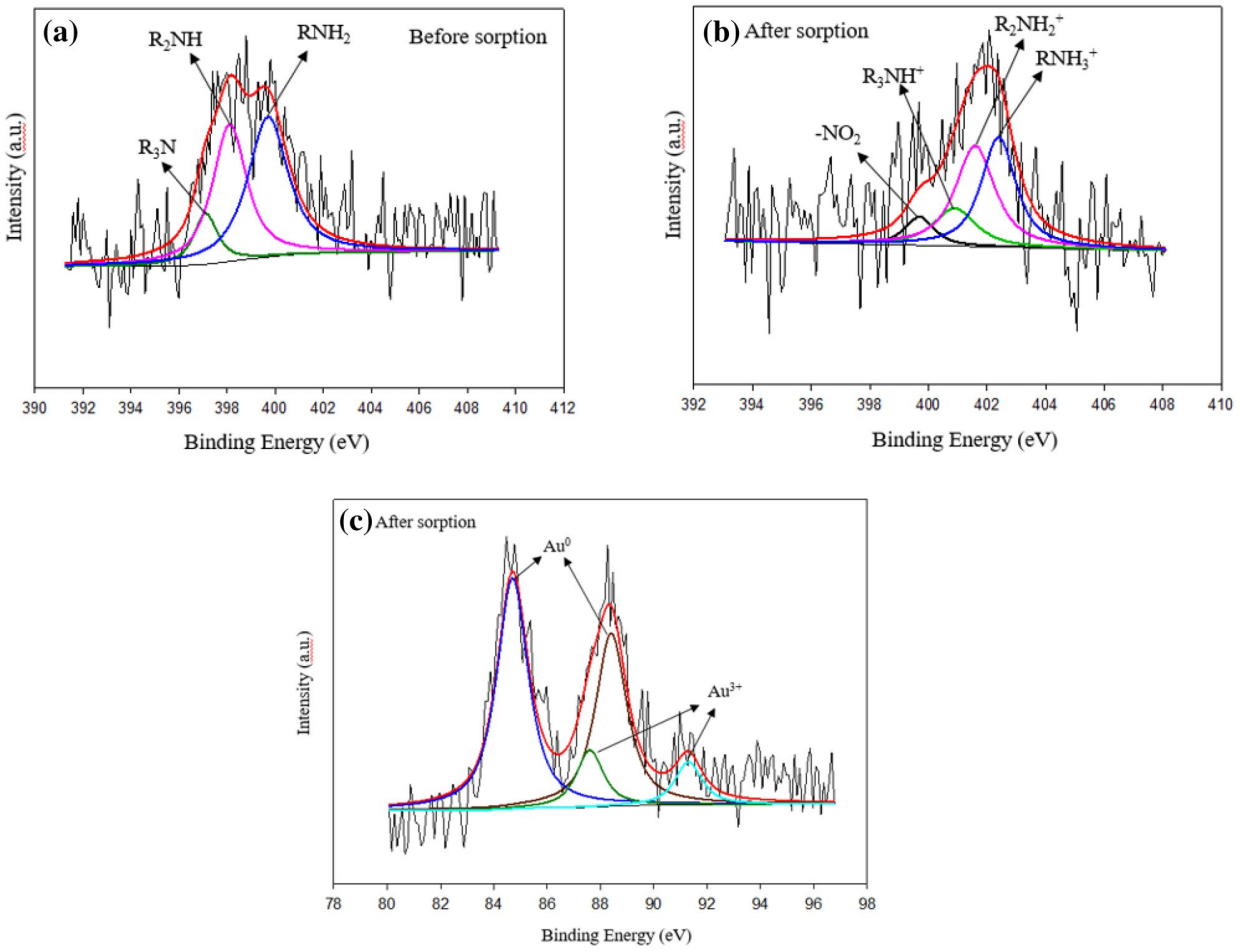
XPS of the capsule (**a**) before sorption, (**b**) after sorption, and (**c**) after sorption of gold.

The XPS was carried out to reveal the adsorption and reduction mechanism of gold with the capsules. The XPS of the capsules before and after the adsorption of gold are displayed in Fig. 8a,b. In the N1s spectra of the capsule, before sorption of gold as shown in Fig. 8a, the peaks at 399.68 eV, 398.10 eV, and 397.12 eV are attributed to RNH_2_, R_2_NH and R_3_N, respectively^59^. After sorption as shown in Fig. 8b, the peaks of N1s spectra appeared at 402.41 eV, 401.61 eV, 400.91 eV and 399.71 eV related to RNH_3_^+^, R_2_NH_2_^+^, R_3_NH^+^ and –NO_2_, respectively^3^. It indicated that the positively charged amines formed after protonation in a strongly acidic solution of gold were the main driving force for the adsorption of anionic AuCl_4_^−^ through electrostatic attraction. The appearance of the peak due to –NO_2_ depicted the oxidation of the amino group during the reduction of gold. Also, Fig. 8c of Au 4f. spectra conformed metallic and ionic gold inside the capsule. The peak at 88.41 eV and 84.71 eV were attributed to Au^0^, whereas the peaks at 87.61 eV and 91.30 eV were due to Au^3+^. Previous studies also supported the adsorption and reduction of gold inside PEI-Alginate composites. From FTIR, XRD, and XPS data, possible adsorption and reduction mechanism of gold with the capsule can be shown as the following equations:

Ionic interaction followed by reduction

7 $$RN{H}_{3}^{+}{Cl}^{-}+Au{Cl}_{4}^{-} \rightleftharpoons \left(R-N{H}_{3}^{+}\right)Au{Cl}_{4}^{-}+ {Cl}^{-}$$

8 $${R}_{2}N{H}_{2}^{+}{Cl}^{-}+Au{Cl}_{4}^{-} \rightleftharpoons \left({R}_{2}N{H}_{2}^{+}\right)Au{Cl}_{4}^{-}+ {Cl}^{-}$$

9 $${R}_{3}N{H}^{+}{Cl}^{-}+Au{Cl}_{4}^{-} \rightleftharpoons \left({R}_{3}N{H}^{+}\right)Au{Cl}_{4}^{-}+ {Cl}^{-}$$

10 $$RN{H}_{2}+ Au{Cl}_{4}^{-}+2{H}_{2}O \to 2{Au}^{0}+R-N{O}_{2}+6{H}^{+}+8{Cl}^{-}$$

## Conclusions

A capsule with a high adsorption capacity was prepared using a simple method for gold recovery. The mechanism analysis showed the ionic attraction of AuCl_4_^−^ with cationic amines and reduction of Au (III) to Au (0) with primary amines. The pH edge experiment revealed an increase in Au (III) sorption but a decrease in Au (I) sorption with increasing the pH. The maximum Au (III) adsorption capacity of the capsule was 3078 mg/g, which was approximately three times that of Au (I). The equilibrium state was attained at 8 h for Au (III) and 4 h for Au (I). The experimental data fit well with the pseudo-second-order kinetic models and Freundlich isotherm models. Thus, the capsule could be evaluated as a high-efficiency adsorbent for the recovery of gold from an acidic solution.

## Data Availability

All data generated or analyzed during this study are included in this published article.
